# Childhood dental caries in context: associations with parental oral health literacy, parenting practices, stress, and socioeconomic status

**DOI:** 10.3389/fdmed.2026.1862989

**Published:** 2026-09-01

**Authors:** Maria Eugênia Domingueti Rabelo Ribeiro, Lara Evangelista Orlandi, François Isnaldo Dias Caldeira, Leandro Araújo Fernandes, Lucas Guimarães Abreu, Murilo César do Nascimento, Daniela Coelho de Lima, Heloisa de Sousa Gomes

**Affiliations:** 1 School of Dentistry, Federal University of Alfenas, Alfenas, Minas Gerais, Brazil; 2 Department of Morphology, Genetics, Orthodontics and Pediatric Dentistry, School of Dentistry at Araraquara, UNESP-São Paulo State University, Araraquara, São Paulo, Brazil; 3 Department of Diagnosis and Surgery, School of Dentistry at Araraquara, UNESP-São Paulo State University, Araraquara, São Paulo, Brazil; 4 School of Dentistry, Federal University of Minas Gerais, Belo Horizonte, Brazil; 5 School of Nursing, Federal University of Alfenas, Alfenas, Brazil; 6 School of Dentistry, Federal University of Goiás, Goiânia, Brazil

**Keywords:** child health, dental caries, literacy, oral health, parent-child relations

## Abstract

**Introduction:**

Dental caries remains a global public health issue influenced by multiple family, psychosocial, and socioeconomic factors, including parental oral health literacy and parenting practices. However, the associations between parental oral health literacy, parental stress, parenting practices, and children's dental caries experience are still not fully understood. Therefore, this cross-sectional study evaluated the associations of parental oral health literacy, parental stress, parenting practices, and socioeconomic status with children's dental caries experience assessed during dental care.

**Methods:**

A cross-sectional study involved children aged 4 to 12 attending the Pediatric Dentistry Clinic at the School of Dentistry, Federal University of Alfenas. Children underwent dental prophylaxis and a clinical examination to assess caries using the dmft/DMFT index. Parents’ oral health literacy was measured with the Brazilian version of the Rapid Estimate of Adult Literacy in Dentistry (BREALD-30), parental stress with the Parental Stress Scale (PSS), and family bonding with the Parenting Practices Inventory (PPI). Statistical analysis was performed using the Kruskal-Walli's test and Poisson regression, with a significance level of 5%.

**Results:**

Among 134 parent–child dyads, children were mostly male (52.2%; *n* = 70; mean age: 7.64 ± 2.21 years), and parents/guardians were predominantly female (88.1%; *n* = 118; age: 29–40 years). Higher parental oral health literacy, more positive parenting practices, older parental age, and older child age were associated with lower dmft/DMFT values, whereas lower family income was associated with higher dmft/DMFT values.

**Conclusion:**

Higher parental oral health literacy was associated with lower DMFT/dmft values in children. Parenting practices, socioeconomic status, parental age, and child age were also associated with children's dental caries experience.

## 1 Introduction

Dental caries, recognized by the World Health Organization (WHO) as a significant global health issue, continues to be one of the most prevalent chronic diseases worldwide (1). In Brazil, the prevalence of dental caries has decreased between the most recent national data collections, conducted in 2003, 2010, and 2023. By 2023, Brazil was classified as having a low prevalence of caries, with dmft/DMFT indices of 2.14 and 1.68 teeth, respectively. However, socioeconomic and demographic disparities significantly influence these outcomes. Notably, there is a stark regional variation in DMFT values, with nearly a 90% difference observed between Brazil's North and Southeast regions (2).

In addition to being a biofilm- and sugar-dependent disease, as described by the Keyes Triad (3), caries can also be affected by secondary or modifying factors, including social, economic, and behavioral issues, as well as the surrounding environment in which an individual lives (4). The study by Qiu et al. (5) further demonstrated that lifestyle and family relationships play a significant role in an individual's vulnerability to caries, highlighting the critical importance of oral health care within the context of family dynamics, daily habits, and psychosocial issues.

One of the concepts that has been more recently debated is oral health literacy, which is defined as an individual's ability to obtain, process, and understand information about oral health, thereby enabling them to make more informed and appropriate decisions (6). A low level of oral health literacy has been linked to lower knowledge and greater difficulty in comprehending instructions and guidance related to oral health care, thus impairing self-care (7–9). Parents play a crucial role in managing their children's general and oral health, and their level of understanding and knowledge may be associated with their children's dental caries experience (8, 10–12).

Beyond oral health literacy, the influence of family dynamics on children's dental caries experience should be considered, particularly in light of the dependent parent-child relationship during childhood. Such dynamics may influence oral hygiene practices and the use of dental care services (10, 13). Studies show that a negative parental relationship and lack of parent–child involvement may influence children's behavior and development (14, 15). Therefore, the influence of this relationship on children's dental caries experience needs to be better understood, especially during childhood, when individuals are developing habits (16). Further studies are needed in this area, as evidence remains scarce.

Previous studies have reported associations between parental oral health literacy and children's dental caries experience (7–9, 12). However, less is known about how parental stress and parenting practices are associated with this outcome within the family context. Therefore, this study was based on the hypothesis that lower parental oral health literacy, higher parental stress, less positive parenting practices, and lower socioeconomic status would be associated with greater dental caries experience, as measured by dmft/DMFT values, in children. To test this hypothesis, we conducted an analytical cross-sectional study with parent-child dyads seeking pediatric dental care at a public university clinic in southeastern Brazil.

## 2 Material and methods

This study was developed following the STROBE Statement- Checklist of cross-sectional studies, and all recommended guidelines were strictly observed throughout the research process.

### 2.1 Study location

This study was conducted in Alfenas, Minas Gerais (MG), Brazil. This municipality is located in the southern region of the state of Minas Gerais, with a total area of 850.446 km², a population of 78,970 inhabitants, and a population density of 92.86 inhabitants/km², with the population predominantly residing in the urban area**.**

### 2.2 Study participants

A cross-sectional study was conducted with a non-probabilistic convenience sample of 153 parents or legal guardians and their respective children, aged 4 to 12 years, who sought care at the Pediatric Dentistry Clinic of the Federal University of Alfenas (UNIFAL-MG), Brazil. Participants were recruited consecutively during routine clinic visits between April and July 2023, and all those who met the inclusion criteria and agreed to participate were enrolled. This study was submitted to and approved by the UNIFAL Research Ethics Committee (CAAE number 57180222.6.0000.5142).

Before data collection began, the parents/guardians and the children were invited, and those who agreed to participate in the research signed a consent form. The adults signed the Informed Consent Form (ICF), while literate children signed the Assent Form (AF), and children who were not literate signed the Clarified Assent Form (CAF). Only one parent or legal guardian per child was invited to participate in order to avoid duplication of information related to the same child. Individuals who did not meet the age range or those who did not fully complete any scale, leaving essential items unanswered, were excluded from the study.

### 2.3 Sample size determination

To determine the sample size for this cross-sectional study, the calculation was based on the following parameters: a finite population (*N* = 220), a tolerable absolute error (0.05), an average proportion of 64.4%, reflecting the prevalence of parental and perceived stress (17–19), and a confidence level of 95%. This approach yielded a minimum required sample size of 136 participants. Additionally, a 10% margin was added to account for potential losses during data collection, resulting in a final target sample size of 150 participants. Based on the parameters used, the estimated statistical power of the study was approximately 80%, which is considered acceptable for cross-sectional observational research.

### 2.4 Data collection

Parents/legal guardians answered questionnaires to assess the sociodemographic and economic profile of each family, parental stress, and perceived stress in the waiting room of the Pediatric Dentistry Clinic.

The child's dental caries experience was also evaluated after professional dental prophylaxis, followed by a clinical examination using a mirror, WHO probe, and clinical tweezers (Golgran, São Caetano do Sul, São Paulo, Brazil), performed by a previously trained and calibrated professional**.** Dental caries was assessed according to the dmft/DMFT index (20).

The calibration of the three professionals responsible for evaluating the children was carried out through clinical examination and classification using the dmft/DMFT index on a group of children from a school in Alfenas, MG. All three professionals evaluated all the children, and the examinations were then compared, with the kappa index calculated among them, resulting in a kappa coefficient of 0.82, considered almost perfect agreement. These children were not included in the study.

#### 2.4.1 General data collection

##### 2.4.1.1 Oral health conditions

The evaluation was based on the dmft/DMFT classification recommended by the WHO (1997) (20), in which the total and disaggregated values of decayed, missing, and filled teeth were recorded for each individual, according to permanent and primary dentition.

##### 2.4.1.2 Socioeconomic and demographic conditions

The sociodemographic condition of each family was assessed using a socioeconomic questionnaire (21), consisting of 16 questions, through which data on the child's and guardian's age, gender, education level, income, and number of children were obtained.

### 2.5 Scale data collection

#### 2.5.1 Parental oral health literacy

To understand the degree of knowledge that parents had about words related to oral health, the Brazilian version of the Rapid Estimate of Adult Literacy in Dentistry (BREALD-30) was used (22). Participants were asked to read 30 words related to dentistry, in increasing order of difficulty. For each word read correctly without hesitation, one point was awarded. Pronunciation errors were considered in cases where words were read slowly, without rhythm, or when it was necessary to repeat the word or any syllable. The final score ranged from 0 to 30 points, with higher scores indicating a higher level of oral health literacy.

#### 2.5.2 Parental stress

To analyze parental stress, the Parental Stress Scale (PSS) (23), validated in Brazil, was used (24). It consists of 16 items, which are answered on a 5-point Likert scale, ranging from 0 = strongly disagree to 4 = strongly agree, with the total score being the sum of all items. For this purpose, the 8 items that have positive connotations (1, 3–6, 11, 15, 16), considered factors related to parental satisfaction, had their scores reversed. The total score ranged from 0 to 64 points. The higher the score, the greater the parental stress.

#### 2.5.3 Parenting practices

Parenting practices were measured using the Parenting Practices Inventory, which is a scale validated nationally (15), to understand how parents interact and relate to their children. This instrument consists of 16 items (reduced version), answered on a 5-point Likert frequency scale (0 to 4), in which the higher the sum, the greater the parent's interaction with the child, and the greater the use of parenting practices. The questions encompass four dimensions of parental involvement with the child: emotional involvement, teaching, discipline, and social aspects, all referring to parental involvement and each with 4 items. The discipline dimension can be considered a dimension of negative practices, so the score obtained in this dimension should be reversed to obtain the instrument's total score.

In the present study, parental stress was treated as a categorical variable. Since the validation study of the scale does not provide a standardized cutoff point for classifying parental stress, the variable was dichotomized based on the sample mean, following the approach previously used by Brito and Faro (24). Participants with scores equal to or below the sample mean were classified as having low parental stress, whereas those with scores above the sample mean were classified as having high parental stress.

### 2.6 Statistical methods

Data was tabulated in Microsoft Excel version 16.72 and analyzed using IBM SPSS Statistics for Windows, version 22.0 (SPSS Inc., Chicago, IL, USA) (25). Double data entry and triple verification were performed to ensure data accuracy. The normality of continuous variables was assessed using the Kolmogorov–Smirnov test. Categorical variables were expressed as absolute and relative frequencies, *n* (%). Non-normally distributed continuous variables were compared using Kruskal–Wallis's test with Benjamini-Hochberg correction for multiple comparisons. Poisson regression models were used to estimate prevalence ratios and 95% confidence intervals. The significance level was set at 5%.

## 3 Results

Initially, 153 pairs were considered eligible; however, 19 pairs were excluded because they had incomplete data for key study variables, which prevented their inclusion in the final analysis. Therefore, 134 pairs and their children were evaluated in the present study.

Within this sample, most respondents were women (88.1%, *n* **=** 118)**,** with age of 33 (29–40) years (Table 1). Regarding the children, 52.2% (*n* **=** 7) were boys and 47.8% (*n* **=** 64) were girls, with a mean age of 7.60 **±** 2.42 years (Table 2).

**Table 1:** Sociodemographic characteristics of parental–child dyads.

Parental Variable	*n* (%)
Parental sex	
Female	118 (88.1%)
Male	16 (11.9%)
Parental age	
Female	33 (29–40)
Male	35.5 (30.5–38)
Monthly family income (Median 25–75 percentile)	
Female	2,000 (1,300–2,775)
Male	2,800 (2,300–3,125)
Monthly family income category	
1 minimum wages	42 (31.3%)
2 minimum wages	70 (52.2%)
3 minimum wages	12 (9.0%)
4 or more minimum wages	10 (7.5%)
Parental’ educational level	
Illiterate or incomplete early childhood education	-
Incomplete high school	25 (18.7%)
Complete high school	30 (22.4%)
Incomplete higher education	63 (47.0%)
Complete higher education	16 (11.9%)
BREALD	24.47 ± 4.45
PSS	23.07 ± 9.48
PPI	48.36 ± 8.21

BREALD, Brazilian version of the rapid estimate of adult literacy in dentistry; PSS, parental stress scale and parenting practices inventory.

**Table 2:** Demographic characteristics of child.

Child variable	*n* (%)
Child's sex	
Female	70 (52.2%)
Male	64 (47.8%)
Child's age (mean and standard deviation)	
Female	7.60 ± 2.42
Male	7.64 ± 2.21
dmft/DMFT score	
Female	4.42 ± 3.34
Male	5.47 ± 3.38
Overall dmft/DMFT index	4.97 ± 3.39

Concerning monthly family income, most families (52.2%, *n* **=** 70) earned up to 2 minimum wages (MW) per month, followed by 31.3% (*n* **=** 42) earning 1 MW, 9.0% (*n* **=** 12) earning 3 MW, and 7.5% (*n* = 10) earning 4 or more MW (Table 1).

Regarding education, no guardians were illiterate or had incomplete early childhood education. Most guardians (22.4%, *n* **=** 30) had completed high school, followed by those with incomplete high education (47%, *n* **=** 63). In addition, 11.9% (*n* **=** 16) had completed higher education, and 18.7% (*n* = 25) had incomplete higher school. Parents**/**guardians had a mean score of 24.47 **±** 4.45 points in the oral health literacy questionnaire (Table 1).

Concerning parental stress, the sample was fairly evenly distributed, with 44.0% (*n* **=** 59) of parents/guardians classified in the high-stress group and 56.0% (*n* **=** 75) in the low-stress group. The mean score for parenting practices was 48.36 **±** 8.21 points (Table 1).

Regarding the children's dental caries experience, the mean number of decayed teeth was 2.0 **±** 5.0 per child, and the overall dmft/DMFT index was 4.97 **±** 3.39 teeth per child (Table 1).

**Figure 1:**
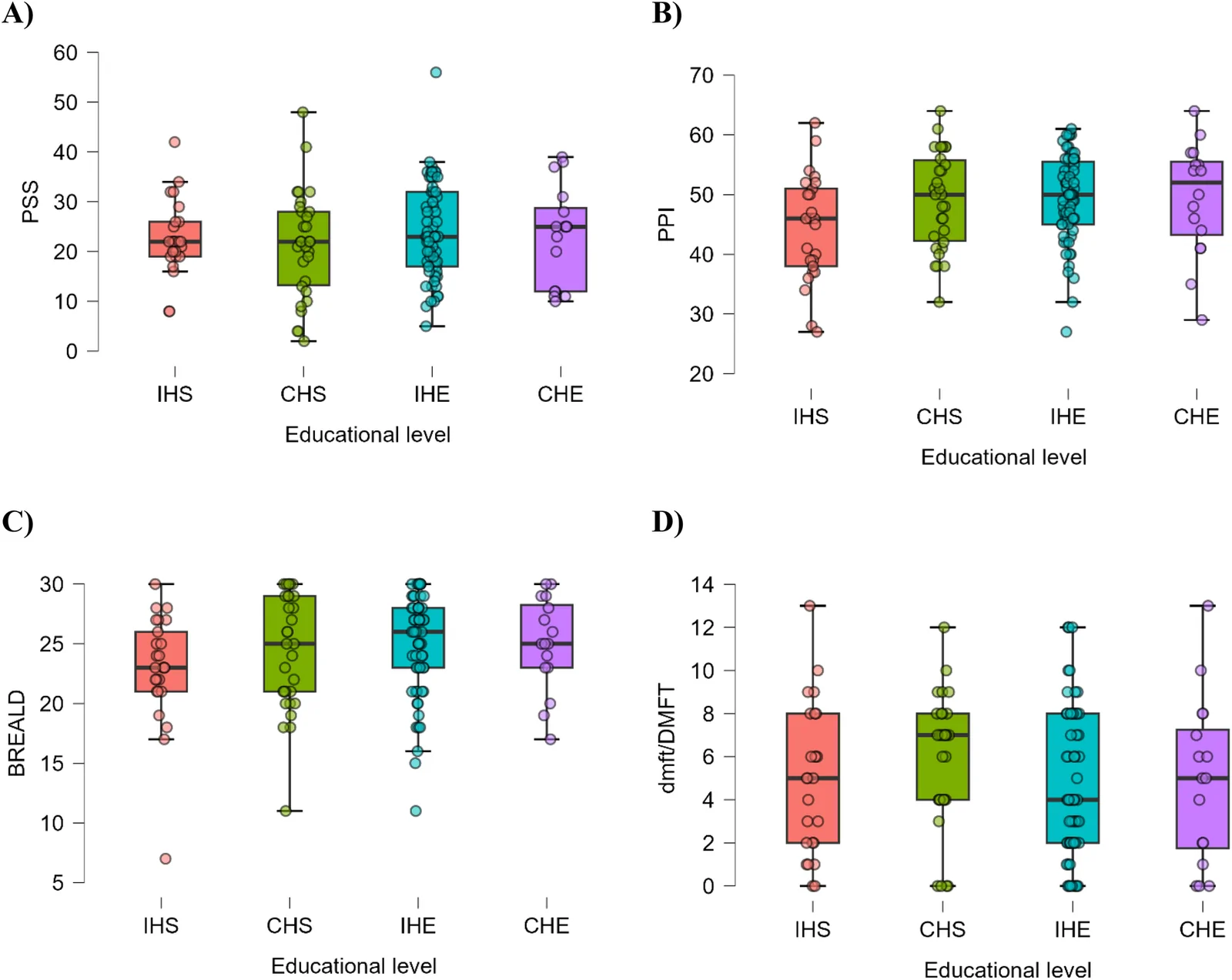
Comparison of parents’ educational levels with PSS, PPI, BREALD and dmft/DMFT. HIS, incomplete high school; CHS, complete high school; IHE, incomplete higher education; CHE, complete higher education; BREALD, Brazilian version of the rapid estimate of adult literacy in dentistry; PSS, parental stress scale and parenting practices inventory. Kruskal–Wallis Test with Benjamini-Hochberg correction. *p* = 0.05.

After Benjamini–Hochberg FDR correction, no statistically significant differences were observed among parental educational levels for parental stress, parenting practices, parental oral health literacy, or children's dmft/DMFT values. Although BREALD-30 scores appeared slightly lower among parents/guardians with incomplete high school, and dmft/DMFT values showed wide variability across groups, these differences were not statistically significant after correction for multiple comparisons (Figure 1).

Poisson regression analysis showed that parental oral health literacy was inversely associated with children's dmft/DMFT scores. Each one-point increase in parental oral health literacy was associated with a 3% lower expected dmft/DMFT score (PR: 0.97; 95% CI: 0.95–0.99; *p* = 0.010). Although this effect appears small when expressed per one-point increase, its interpretation becomes more meaningful across broader score differences. For example, a 5-point increase in parental oral health literacy corresponds to an estimated 14% lower expected dmft/DMFT score, whereas a 10-point increase corresponds to an estimated 26% lower expected dmft/DMFT score (Table 3).

**Table 3:** Multivariable Poisson regression model evaluating the association between parent's oral health literacy and number of decayed, missing, and filled teeth in children in a model controlled for demographic and psychosocial variables.

Variable	Prevalence ratio (PR)	95% confidence interval	*p*-value
Parent's oral health literacy (per 1-point)	0.97	0.95–0.99	**0** **.** **010**
Child's sex			
Female		Ref.	
Male	1.18	0.95–1.45	0.117
Child's age (years) (per 1-year)	0.93	0.89–0.98	**0** **.** **006**
Family income			
1 minimum wages	1.34	0.69–2.60	0.388
2 minimum wages	1.56	0.88–2.78	0.125
3 minimum wages	2.00	1.00–3.96	**0** **.** **047**
4 or more minimum wages		Ref.	
Parent's schooling			
Primary complete	0.85	0.54–1.51	0.908
Secondary complete	1.20	0.74–1.66	1.109
High school	0.90	0.62–1.30	0.904
University		Ref.	
Parent's stress			
Low	1.03	0.82–1.29	0.767
High		Ref.	
Parent's practices (per 1-point)	0.97	0.96–0.99	**0** **.** **003**
Parent's sex			
Female		Ref.	
Male	1.22	0.90–1.66	0.198
Parent's age (years) (per 1-year)	0.98	0.97–0.99	**0** **.** **008**

Ref., Levels of reference; Poisson Regression (*P* < 0,05).

Bold numbers indicate statistically significant differences in the parameter analyzed.

Child age was also inversely associated with dmft/DMFT scores. Each additional year of age was associated with a 7% lower expected dmft/DMFT score (PR: 0.93; 95% CI: 0.89–0.98; *p* = 0.006). Regarding socioeconomic status, children from families with a monthly income of up to three minimum wages had a higher expected dmft/DMFT score compared with those from families with an income of four or more minimum wages (PR: 2.00; 95% CI: 1.00–3.96; *p* = 0.047).

Parenting practices were also associated with children's dental caries experience. Greater parent–child involvement was associated with a lower expected dmft/DMFT score, with each one-point increase corresponding to a 3% lower expected dmft/DMFT score (PR: 0.97; 95% CI: 0.96–0.99; *p* = 0.003). When expressed across broader score differences, a 5-point increase in parent–child involvement corresponds to an estimated 14% lower expected dmft/DMFT score, whereas a 10-point increase corresponds to an estimated 26% lower expected dmft/DMFT score.

In addition, parental age was inversely associated with children's dmft/DMFT scores. Each additional year of parental age was associated with a 2% lower expected dmft/DMFT score (PR: 0.98; 95% CI: 0.97–0.99; *p* = 0.008). When interpreted over a 10-year difference in parental age, this corresponds to an estimated 18% lower expected dmft/DMFT score (Table 3).

## 4 Discussion

In this study, some characteristics of the guardians were associated with the child's dental caries experience, such as age, monthly family income, oral health literacy, and parenting practices. As for the children, age was also a factor related to the presence of decayed, missing, and filled teeth.

There was an association between parents' oral health literacy and the dmft/DMFT index of their children, which is consistent with other studies that also found a higher prevalence of decayed, missing, and filled teeth in children of parents with lower literacy levels (26–28). Additionally, a 5-point increase in oral health literacy was associated with an approximately 14% reduction in the number of decayed, missing, and filled teeth in children, suggesting a potentially relevant protective effect (PR = 0.86). This can be explained by the greater difficulty of parents with low levels of oral health literacy in understanding instructions and guidance, as well as the real importance of performing oral health care, impairing children's self-care (7–9, 12). Since parents are responsible for their children's health care, low knowledge is often associated with less concern about seeking dental care and performing oral hygiene, resulting in a higher number of decayed, missing, and filled teeth among children (8, 10, 11).

Parenting practices were associated with the number of decayed, missing, and filled teeth in the sample, as those with greater parental involvement had a lower dmft/DMFT index. Each 5-point increase in parental practices was associated with an approximately 14% reduction in the outcome. This was also found in other studies, which showed that parents' lifestyle influences their children's dental caries experience (13, 29, 30). However, according to the literature, no studies in dentistry have used this scale of parenting practices to evaluate this relationship, highlighting the relevance of the present study and suggesting the need for more studies to better understand this relationship, in which parenting practices can have a protective effect on children's oral health, including dental caries and the early loss of primary and permanent teeth.

In families with low socioeconomic status, children exhibited a higher dmft/DMFT index, a finding also seen in other studies. Lower monthly income can contribute to a higher incidence of dental caries for various reasons, including the adoption of inadequate habits caused primarily by a lack of access to knowledge, food, and health care (31–35). Another study showed that individuals from a higher social class had lower caries experience (36), and parents with better professional situations also had better oral health (37). In the present study, this association was observed when comparing families with an income of up to 3 MW to those with an income of 4 or more MW.

Another characteristic of the guardian that was observed to be associated with the child's dmft/DMFT index was the young age of the guardian. This factor was also considered a possible factor that may have an influence on the development of caries (30), with children of younger guardians exhibiting a higher caries index, in which each 5-year increase in parental age was associated with a 10% reduction in the outcome (PR = 0.90). In the study by Mattila et al. (30), the focus was solely on the age of the mothers, while in the present study, the questionnaires were answered by the guardians, among whom the majority were mothers.

A study showed that older parents tend to have greater involvement with their children, engage in more parenting practices, and exhibit more patience and sensitivity to children's needs (38), which could influence behavior and care regarding oral health. Additionally, younger parents are more likely to have children with externalizing problems, which are related to hyperactivity, impulsivity, and aggression, fostering conflicts and hindering parent–child relationships, potentially affecting health care as well (39).

No association was found between stress from parenting and the child's dental caries experience. There are no studies in literature specifically addressing this topic. The literature highlights only studies assessing the relationship between parental stress and the child's general development and well-being, without being directly associated with oral health (40, 41). Additionally, some studies analyze family behavior concerning the child's behavior, anxiety, and fear during dental care (14, 15). To confirm this result, more studies on this relationship are needed to verify whether there is no interaction between parental stress and the child's dental caries experience.

Regarding the children, those who were younger had a higher dmft/DMFT index. Each 5-year increase in child age was associated with a 31% reduction in the number of decayed, missing, and filled teeth (PR = 0.69), which differs from studies showing that dental caries tends to increase its prevalence with age, as it is a condition that has a cumulative process and depends on time to evolve if no intervention is performed (35, 42, 43). The inverse association between age and dmft/DMFT should be interpreted within the context of this clinical sample. Younger children attending the university clinic may have presented a greater burden of untreated caries in the primary dentition. Therefore, this result is specific to the present sample and should not be generalized as a broader epidemiological pattern.

### 4.1 Limitations and strengths

A strength of the present study is that it was conducted using *a priori* sample size calculation, which supported the adequacy of the sample for the proposed analyses. In addition, standardized data collection procedures were adopted, including double data entry and triple verification, improving the reliability of the database. The study also included multivariate analysis, allowing the evaluation of associations between parental oral health literacy, parenting practices, parental stress, socioeconomic factors, and children's dental caries experience. Despite these strengths, some limitations should be acknowledged. First, the study has limitations regarding representativeness, as the sample was restricted to children seeking care at a public university clinic. Therefore, the findings cannot be generalized to the general population and should be interpreted as applicable only to children who attended the clinic where the research was conducted. The Poisson regression models were not accompanied by a reported assessment of overdispersion or robust standard errors. Another limitation of the present study is the combined use of the dmft and DMFT indices among children aged 4 to 12 years. Although this approach was adopted to estimate overall dental caries experience across dentitions, it does not distinguish caries burden in primary and permanent teeth. Therefore, possible dentition-specific differences may have been obscured. Future studies should analyze dmft and DMFT separately, particularly in samples including children in the mixed dentition stage. Because of the cross-sectional design of the present study, causal relationships between the investigated variables cannot be established. Therefore, the findings should be interpreted as associations between parental oral health literacy, parenting practices, parental stress, socioeconomic factors, and children's dental caries experience, rather than evidence of cause-and-effect relationships.

## 5 Conclusion

It is possible to conclude that parental oral health literacy is related to children's oral health condition, specifically to the mean number of decayed, missing, and filled teeth. Higher parental literacy was associated with lower dmft/DMFT values in children. Additionally, factors such as parenting practices, socioeconomic status, guardian age, and child age were also related to the presence and number of decayed, missing, and filled teeth. Regarding parental stress, no association with the child's dmft/DMFT index was observed.

## Data Availability

The raw data supporting the conclusions of this article will be made available by the authors, without undue reservation.
